# Assessing and comparing early warning signal performance in spatially-structured systems

**DOI:** 10.1371/journal.pone.0332695

**Published:** 2025-10-06

**Authors:** George E. Robinson, Graham M. Donovan

**Affiliations:** Department of Mathematics, The University of Auckland, Auckland, New Zealand; Università Cattolica del Sacro Cuore Sede di Piacenza e Cremona Facoltà di Economia: Universita Cattolica del Sacro Cuore Facolta di Economia e Giurisprudenza, ITALY

## Abstract

Critical transitions occur when a system undergoes a sudden shift from one state to another. Early warning signals (EWS) are indicators that can be used to potentially anticipate critical transitions in such systems, which may be temporal or spatio-temporal. Temporal systems are those whose state varies over time, whereas spatio-temporal systems also vary over a spatial domain. While temporal EWS can be applied to spatio-temporal systems by averaging over the spatial domain, spatially-informed EWS should, in principle, be able to outperform their temporal counterparts by making use of the additional spatial information. We seek to understand how EWS for spatial systems compare to those used for temporal systems. To facilitate comparison, we explore how EWS performance is measured. We use the strength of EWS trends, quantified using Kendall’s *τ*, as a proxy for performance. Other factors, such as robustness to choices of parameters used for detrending, statistical significance, and agreement with expected EWS behaviour, are considered. This assessment of EWS based on these factors enables an informed comparison and decision regarding which signals to apply to different systems for potential indications of critical transitions. We find that while spatially-informed EWS generally offer improved performance over temporal EWS for the example systems studied, we find the choice is system specific.

## Introduction

Tipping points, also known as critical transitions or regime shifts, occur when a system undergoes a relatively sudden shift from one (stable) state to another [1]. Many phenomena from different fields display sudden shifts, including climate and ecological systems such as the shift at the end of the Younger Dryas event ‘where the Arctic warmed $7^{\circ }$C in 50 years’ [2] and the desertification of North Africa from a savanna-like state with lakes to a desert [2,3]. Physiological systems also display abrupt transitions. These include asthma attacks, where airways display bistability between nearly closed and open states in spatially distributed patterns known as clustered ventilation defects [4–6]. Another example of physiological phenomena considered to display tipping points are epileptic seizures [7]. Often these shifts are the result of small changes in the underlying conditions of the system, and the consequences of the transitions are undesirable. Furthermore, restoring the underlying conditions of some systems to values which preceded the transition do not necessarily revert the system back to its original state. As critical transitions are present in a rich variety of systems and are not easily reversible, we would like to be able to predict critical transitions in advance, allowing for potential mitigation.

Early warning signals (EWS) are quantitive metrics derived from data that have the potential to predict critical transitions. Temporal EWS are metrics which have the potential to anticipate critical transitions in temporal systems. Spatial EWS are indicators which have the potential to anticipate critical transitions in systems which have spatial structure. Spatial EWS are of interest because spatio-temporal systems provide a richer data set; this means that for each point in time there is spatially distributed data from which to compute indicators of potential critical transitions, which may allow for better predictions of critical transitions [8]. It is important to acknowledge that while EWS have the potential to predict critical transitions, they are not definitive predictors.

Temporal EWS can also be applied to spatio-temporal systems by applying the EWS to a temporal data set derived from the spatio-temporal data, for example the spatial mean of a system. Hence, temporal EWS can be compared to spatio-temporal EWS (for spatio-temporal systems). This is of interest as tipping points in temporal systems are well understood compared to tipping points in spatio-temporal systems, and have been characterised to a relatively small number of underlying mechanisms [9,10]. However, the temporal data sets derived from spatio-temporal systems disregard the changes in the spatial structure and reduce the additional information of a system captured in spatio-temporal data sets. This results in the following question: how do spatial and multivariate EWS, which take advantage of spatio-temporal data, compare to temporal EWS applied to reduced systems derived from spatio-temporal systems?

In this paper we address this question by computing EWS for spatial data sets and compare EWS by quantifying trends in the signals using Kendall’s *τ*. Furthermore, we analyse the robustness of Kendall’s *τ* against detrending parameters used when preparing data sets and computing EWS. We extend the existing comparison of EWS to include the eigenvalues of the covariance matrix [11] which has previously only been compared to spatial variance, spatial skewness, and spatial correlation but not their temporal counterparts. A significant subject of this current work is measuring the performance of an EWS, as this facilitates their comparison. At present, different methods and quantifications are sometimes used, making comparison difficult. Kendall’s *τ* rank correlation coefficient is often used to quantify the trend of an EWS over a given interval [6,11–15]. Hence, in this paper we explore how trends of the signals are used as a proxy for their performance.

We use elements of the methodologies presented by Dakos et al. [12] and Kéfi et al. [16] to compare spatially informed and temporal EWS for spatio-temporal systems. Previously Dakos et al. [12] developed a methodology for applying temporal EWS to time series data. Furthermore, Kéfi et al. [16] developed a methodology for applying spatial EWS to spatio-temporal systems. Following these approaches for applying EWS is the *Spatial early warning signs* R package, the use of which is discussed in Génin et al. [17]. A summary of the existing available software tools for estimating spatially informed and temporal EWS can be found in ref [18].

We apply our analysis of EWS to three synthetic data sets and one empirical data set. The first model is intrinsically discrete, in which using a system of coupled lattice points naturally describes the behaviour of the system. Hence the first model we use is a stochastic lattice dynamical system (SLDS). The second model is obtained by approximating the original model and taking the continuum limit to obtain a reaction-diffusion equation with added noise. This second model is a stochastic partial differential equation (SPDE). Often in practice when studying spatial EWS, synthetic data sets are generated using SLDS obtained by discretising reaction-diffusion equations or spatially extending a one-dimensional dynamical system by defining a lattice of state variables and including diffusion between neighbouring lattice sites using the discrete Laplacian; both approaches result in the same general form of SLDS [11,13,16,19]. Hence we discretise the derived stochastic reaction-diffusion equation to obtain an SLDS model with similar structure to models which have previously been used to study spatial EWS. The third model is a spatially extended dynamical system from ecology with added noise. The empirical data set used contains a transition from savanna to woodland in the Serengeti ecosystem along a spatial (rather than temporal) gradient which is correlated with an increase in annual rainfall. This data set has been previously used to study spatial EWS by Deb and Dutta [20] and can be found in a previous study by Eby et al. [21]. From these synthetic and empirical data sets we analyse how the EWS behave for the different synthetic data sets. This analysis allows for informed EWS choice. Additionally, we deduce that EWS can respond differently, in important ways, to different models of the same system.

## Methods

### Early warning signals

#### Temporal EWS.

We consider four generic temporal EWS which are the variance, skewness, and autocorrelation of a time series data set (the autocorrelation is measured using both the autoregression coefficient at-lag-1, and the autocorrelation function coefficient). We estimate the variance and skewness using backwards rolling windows of length *W*_*s*_ on detrended temporal data sets. We define *W*_*s*_ as the number of data points contained within the window. Backwards rolling windows estimate the signal at time *t* using the previous *W*_*s*_ data points - assuming that the previous states of the system can be used as approximation for an ensemble at time *t*. We estimate the autocorrelation of the time series using the autocorrelation function at lag 1 and also fit an autoregressive model of order 1 between the the variables *X*_*t*_ and *X*_*t*−1_ over a backwards rolling window of length *W*_*s*_. We take the indicator at time *t* as the autoregressive coefficient estimated from the autoregressive model of order one computed using the values in the backwards rolling window.

#### Spatially-informed EWS.

The five spatially-informed EWS we consider are the spatial variance, spatial skewness [22], spatial correlation [19], largest eigenvalue of the covariance matrix, and the percentage that the largest eigenvalue accounts for of the total variation [11]. We compute the spatial variance and spatial skewness using the spatial information available in a given snapshot at time *t* using detrended spatio-temporal data sets. Both of these EWS are typically multivariate, as permuting the lattice elements does not result in a different value of the indicator at time *t* [16]. It should be noted that this is not true in general. For coarse grained spatial data these indicators are no longer invariant to permutations of the lattice sites [23]. We compute the spatial correlation using Moran’s I at lag-1 for elements that share an edge (and not a vertex). Spatial correlation is a true spatial EWS as it is not invariant under permutations of the lattice sites. This is because permuting the lattice sites results in a different adjacency matrix *W* used in the calculation of Moran’s I.

The eigenvalues of the covariance matrix can be used as indicators of critical transitions as Chen et al. have shown that under certain conditions the largest eigenvalue and the proportion that the largest eigenvalue accounts for the total variation increase as the system approaches a critical transition [11]. The unbiased empirical covariance matrix *S* of an N×N lattice of state variables *X*_*i*,*j*_ at time *t* indexed by i,j=1,…,N, is estimated using a backwards rolling window as follows. Let the state variables be re-indexed using linear subscripts where $i,j=1,\ldots ,N^{2}$, then the elements of the covariance matrix can be estimated as

$$S_{jk}=\frac{1}{n-1}\sum_{i=1}^{n}(X_{ij}-{\overset{―}{X}}_{j})(X_{ik}-{\overset{―}{X}}_{k})$$ (1)

where *S*_*jk*_ is the covariance of the variables $X_{j}\text{ and }X_{k}$. The number of snapshots in the rolling window is *n*, and ${\overset{―}{X}}_{j},{\overset{―}{X}}_{k}$ are the averages of the variables $X_{j},X_{k}$ within the window. We compute the unbiased covariance matrix using MATLAB’s cov() function. The eigenvalues of the estimated covariance matrix were calculated using MATLAB’s eig() function. The eigenvalues of the covariance matrix are multivariate EWS. A spatial permutation of the lattice sites does not affect the covariance of the variables *X*_*j*_ and *X*_*k*_, hence the covariance matrix *S* remains unchanged, and therefore the eigenvalues of the matrix are invariant to spatial permutations of the lattice elements.

### Detrending

Spatio-temporal data sets and temporal data sets are often detrended prior to computing EWS. For signals computed using temporal averaging over a window such as the variance for temporal data sets or the eigenvalues of the covariance matrix for spatio-temporal data sets, the mean of the data must be approximately stationary. To ensure the mean is approximately stationary, we must detrend the data, as ‘non-stationarities in the mean can cause false indicators of impending transitions’ [12]. We detrended temporal data using linear detrending, and detrended spatio-temporal data sets uniformly by removing the spatial mean, meaning we subtracted the same value from each lattice site at a given time to obtain the spatio-temporal residuals which are then used to compute spatially informed EWS. Consequently, as we detrend spatio-temporal data sets by uniformly removing the spatial mean of each snapshot and therefore neither a window size or smoothing bandwidth is required, then several spatial/multivariate EWS do not need to be tested for their robustness to detrending parameters, and are therefore invariant to choices of *W*_*s*_.

### Measuring the performance of EWS

We use Kendall’s *τ* to measure the strength of a trend in an EWS. Consequently, we consider the magnitude of the trend in the signal as a partial proxy for its performance. Additionally, we also consider the significance and robustness of the signals. Kendall’s *τ* is computed from the EWS and the bifurcation parameter for synthetic data sets produced from models where a known underlying bifurcation parameter changes linearly with time, such as the data sets we have used where the bifurcation parameter is proportional to εt, where *ε* is the time scale separation of the fast and slow dynamics. In empirical data sets where it is not known whether there is a single underlying parameter which is changing and is responsible for the transition, the correlation is between the signal and time (assuming an unmeasurable underlying bifurcation parameter changing linearly with time). Less commonly, the correlation can be computed between the signal and a spatial index of the data. This is the case for data sets where the system responds to an underlying driving variable which changes along a spatial gradient, such as rainfall in an ecosystem.

In existing methodologies for applying EWS to spatio-temporal or temporal data sets, significance testing of the trends takes place in order to determine that trends identified in the signals are used not due to chance alone [3,12–14,16]. In these papers the authors use parametric tests to identify statistically significant trends. This is achieved by creating surrogate data sets using various methods to then determine the probability that the trend in the data is not due to chance alone. We have used the non-parametric modified Mann-Kendall test as proposed by Hamed and Rao [24] to determine statistical significance of a signals trend. The modified Mann-Kendall test accounts for autocorrelation in the data, which occurs in EWS computed using sliding overlapping windows. Chen et al. show that the non-parametric modified Mann-Kendall test and parametric significance testing yield similar results in the case of low-dimensional systems with Gaussian noise [25]. The benefit of the non-parametric test is that surrogate data sets are not necessary to estimate the statistical significance. This increases the computational efficiency and reduces the additional choice required when implementing a parametric method of choosing an appropriate method to generate surrogate data sets. Details on the modified Mann-Kendall test can be found in S2 Appendix.

EWS and their trends are sensitive to choices of parameters used to compute them; consequently the window size is a crucial methodological choice. As the window size is a crucial methodological choice, we perform a sensitivity analysis (similar to refs [3,12,14,15]) to understand how Kendall’s *τ* is influenced by these choices. In this sensitivity analysis we compare Kendall’s *τ* to changes in the window size. Furthermore, as an additional metric/consideration of EWS performance, we also consider the proportion of window sizes that produce significant trends. Hence, a well-performing EWS should be robust to a large range of parameter choices used when computing the signals.

In addition to assessing the significance and robustness of trends identified in EWS, we further improve our methodology for assessing trends in EWS by repeating simulations of each model. For each model we simulate 100 data sets. For each realisation we compute the EWS, from which we compute the Kendall’s *τ* values and their associated *p*-value using the Modified Mann-Kendall test. Repeating simulations allows us to obtain an estimation of the spread of the trends, in addition to how they respond to changes in the window size. Furthermore, repeating simulations of the models improves the significance analysis of the trends. This is because we use repeated realisations to obtain the percentage of significant trends across all realisations of the data rather than relying on the *p*-value from a single realisation of each system.

## Models and data sets

We use two physiological models and one ecological model that undergo critical transitions to generate data sets which we use to compare EWS. Furthermore, we also compare EWS for a real-world empirical ecological data set to improve the strength of our results. The physiological models describe the behaviour of coupled terminal airways of asthmatic lungs. When an asthma attack occurs, airways constrict in clusters. This airway constriction, known as clustered ventilation defects, occurs as spatially distributed patterns which vary (at least partially) from event to event. Therefore, the abrupt shift from homogeneous to clustered ventilation distributions is a spatial tipping point [4]. The ecological model describes a biomass under harvesting which undergoes a critical transition from a abundant state to an over-exploited state. The empirical data set we have used contains spatially distributed vegetation data from the Serengeti-Mara ecosystem [20,21]. The data set contains a binary representation of either woodland or grassland, as savanna transitions to forest across a spatial (rather than temporal) gradient. This spatial gradient is correlated with a change in mean annual rainfall across the region.

### Model details.

The first model [6] is a stochastic lattice dynamical system (SLDS) where the state variables are airway radii *r*_*i*,*j*_, indexed on an N×N lattice ℒ, with bifurcation parameter κ which describes the constricting force due to smooth airway muscle activation. Here we do not review the physiological modelling but refer the reader to ref [6], which is in turn an empirical reduction of ref [5]. The second model is a stochastic partial differential equation (SPDE) derived from the intrinsically discrete SLDS model first described in ref [6]. This SPDE model is formulated to resemble the reaction-diffusion models with additive white noise commonly used to generate synthetic spatio-temporal data used in the study of spatial EWS. The third model is a spatial ecological model which describes a spatially distributed biomass under harvesting, with bifurcation parameter *c* which describes the harvesting rate. This spatial ecological model has been widely used to study spatial EWS and is derived from a well-studied one-dimensional ecological model with alternative stable states [11,12,19,26]. The one-dimensional model describing the total biomass was adapted by including a dispersion term given by the discrete Laplacian and by introducing a multidimensional white noise process, $\sigma dW_{i,j}$. In each model we use the stochastic fast-slow framework described in [10] where the bifurcation parameter is the slow state variable whose dynamics evolve linearly with time and are controlled by the timescale separation parameter *ε* where 0<ε≪1. Full descriptions of the three models can be found in S3 Appendix.

Each stochastic fast-slow system we use undergoes a bifurcation of the quasi-static equilibrium in the singular limit (ε→0) with zero noise (σ=0). We generate data sets along a gradient of the bifurcation parameter which exceeds the the location of the bifurcation of the fast subsystem in the singular limit. This is because in the non-singular limit with noise, the tipping location is not necessarily equal to the location of the bifurcation of the fast subsystem, but can occur prior to or after the bifurcation of the fast subsystem. This early/delayed tipping relative to the bifurcation of the fast subsystem can occur for a variety of reasons. Noise induced tipping can occur before the bifurcation and the timescale separation can cause the tipping event to be delayed over a long time period; effectively delaying the shift of the system to an alternative state. Hence the tipping points for the non-singular limit systems with non-zero noise are hard to find analytically due to changes in the parameters *ε* and *σ*.

### Data subset selection.

For reasons we have just discussed, the location of the tipping points are random variables, and therefore have associated distributions. This is important because we use repeated simulations of the models to produce multiple realisations of the data sets; as this allows us to improve our understanding of the significance and spread of the trends in the signals. By understanding how the tipping locations are distributed it allows us to: a) generate data sets which always contain the tipping point for choices of the noise and time scale separation, and furthermore b) select intervals of the EWS for trend analysis that always precede the potential tipping locations.

**Fig 1:**
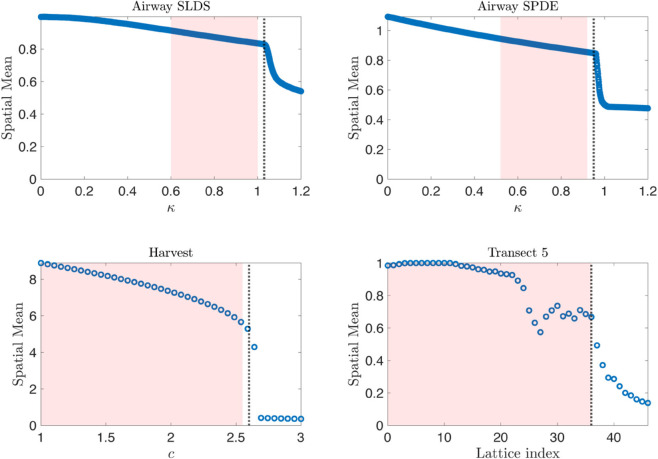
Spatial mean of each model with location of observed tipping points. The spatial mean of each model is plotted against the underlying bifurcation parameter. In each image, the red shaded region indicates the subsection of the bifurcation parameter range used to assess the trends of the EWS computed from the spatial and temporal data sets. The vertical black dashed line indicates the typically observed tipping point in each system for the parameter values that we have used.

The actual location of the tipping points in the synthetic data sets are estimated from observing where the sudden shift in the spatial mean occurs. Previously Dakos et al. [3] compute and assess EWS for historical empirical data containing transitions. In these data sets the tipping point has already occurred and must be chosen from observing the data set; likewise we estimate the location of the tipping points by observing the spatial mean of our systems. In the airway SLDS model the tipping point is observed to typically occur at κ≈1.03, in the airway SPDE model the tipping point is observed to typically occur at κ≈0.95, which can be seen in Fig 1. These observed tipping points occur for a white noise term where σ=0.01, on a 20×20 lattice, with a time scale separation of ε=0.01. For the harvesting model, we observed the tipping point to typically occur at c≈2.6 for σ=0.1, for a 100 × 100 lattice with a time scale separation of ε=0.001.

Estimating the lower bound of the distribution of tipping locations provide an upper bound for the subsection of the EWS used for trend analysis. Other factors must also be considered when selecting the lower bound of the subsection of the EWS used to compute the trend. For example, the airway data sets are generated from models for which the underlying eigenstructure is known [5] and does not display critical slowing down (CSD) for all bifurcation parameter values before the transition. In these models, the leading eigenvalue of the fast subsystem does not increase monotonically but rather decreases before monotonically increasing after approximately κ=0.6. Hence, for these models we expect to indirectly capture CSD using the EWS in the region of the data where the leading eigenvalue increases monotonically. Therefore, we compute Kendall’s *τ* for the EWS associated with the subsection of the bifurcation parameter range where the leading eigenvalue is increasing and prior to the tipping point. This is not the case for the harvesting model, hence any subset of the EWS preceding the transition should provide indication of CSD.

The subsets of the data sets used for EWS analysis are those which lie in the following intervals of the underlying bifurcation parameter: κ∈[0.6,1], κ∈[0.52,0.92], c∈[1,2.55] for the SLDS, SPDE, and harvesting model respectively. For the empirical ecological data we computed the trend using all data points prior to the transition. Furthermore, we prepared the raw transect data according to the methods described by Eby et al. [21]; resulting in a spatio-temporal data set of forty-seven 50 × 50 lattices. The subsets used for analysis for each model are indicated by the regions of the data sets shaded red in Fig 1.

Synthetic data sets are produced from the models numerically using the Euler-Maruyama method. For each model, a SLDS describes the fast subsystem and the underlying bifurcation parameter is incorporated as a state variable in the slow-subsystem. The time scale separation between the fast and slow subsystems for the airway models is ε=0.01; for the harvesting model we use a timescale separation of ε=0.001. In each case the models we solve numerically are SLDS; data sets for the reaction-diffusion SPDE model were obtained by first discretising to obtain a SLDS. In each model the initial conditions are the unperturbed homogeneous equilibrium of the fast subsystem. We used periodic boundary conditions. For the airway models we used a white noise with standard deviation σ=0.01 and σ=0.1 for the harvesting model. All data sets are produced over ranges of the underlying bifurcation parameters such that the critical transitions of the systems are always present in the data sets.

### Data resolution.

In practise, observations of real-world systems are limited by factors such as costs or sampling methods - often resulting in small data sets with low and potentially irregular frequency. Under the fast-slow framework, the temporal resolution of the raw data produced by the Euler-Maruyama method is high. Therefore we down-sampled the raw data to better reflect data sets observed from real-world systems. For the airway models we down-sampled the raw data to data sets containing one thousand 20 × 20 lattices. For the harvesting model we down-sampled the raw data to data sets of forty 100 × 100 latices. It is important to note that the frequency of the observations should be chosen to be shorter than the characteristic time scales of the slowest return rate of the system [2,12,27].

The range of window sizes we use to assess the robustness of signals is dependent on the properties of the data sets. For the airway data sets we let the window size vary from 5 to 50 percent of the entire length of the data set. For the ecological data sets we let the window size vary from 25 to 50 percent. In the harvesting model data set containing only 40 spatial snapshots, a small window size would contain too few points to accurately estimate the values of the EWS. Hence, for low-temporal-high-spatial-resolution data as is commonly found in empirical ecological data sets we must be careful when detrending data and computing EWS from windows containing insufficient data points. Further work could explore how the error introduced using small window sizes when computing EWS affects the statistical significance and robustness of the trends.

## Results

**Fig 2:**
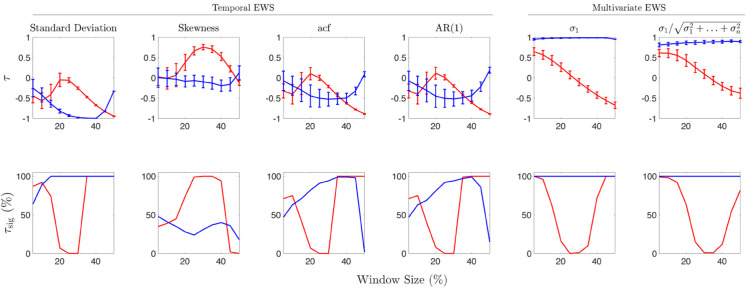
Kendall’s *τ* against *W*_*s*_ for airway model data sets. (Row 1) Mean Kendall’s *τ* values for airway models as the window size is varied from 5 to 50 percent with standard deviation given by error bars. Each panel displays the sensitivity of *τ* for the SLDS model (red) and the SPDE model (blue). (Row 2) Percentage of statistically significant Kendall’s *τ* values as the window size is varied.

**Fig 3:**
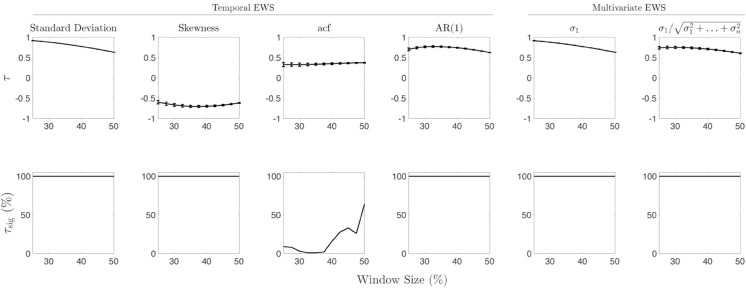
Kendall’s *τ* against *W*_*s*_ for harvest model data sets. (Row 1) Mean Kendall’s *τ* values for harvest model as the window size is varied from 25 to 50 percent with standard deviation given by error bars. Each panel displays the sensitivity of *τ* for the harvest model. (Row 2) Percentage of statistically significant Kendall’s *τ* values as the window size is varied.

**Fig 4:**
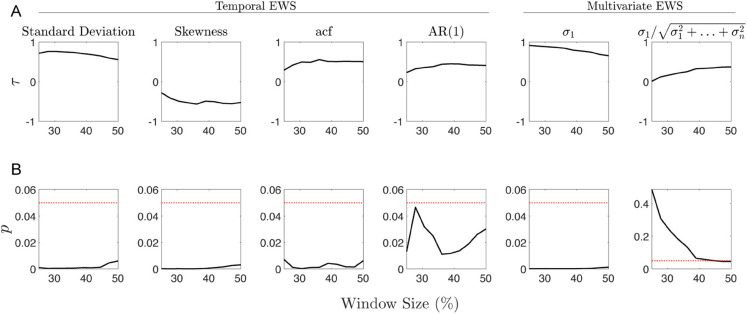
Kendall’s *τ* against *W*_*s*_ for Transect-5. (A) Kendall’s *τ* values for Transect-5 data as the window size is varied from 25 to 50 percent. (B) *p*-values of Kendall’s *τ* value as the window size is varied.

We computed Kendall’s *τ* to quantify the trends of various spatial and temporal EWS, for multiple realisations of several models that undergo tipping. These values alongside other considerations act as a proxy for the performance of an EWS. Using this measure of EWS performance enables us to compare the EWS. Additional factors that have been considered when evaluating EWS are the robustness and statistical significance of the trends. For clarity, in the following two sections we present the results for the airway data sets and ecological data sets separately. Figs 2 to 4 display the robustness, statistical significance, and magnitude of the trends as well as their directions for the different data sets. Table 1 contains the mean and standard deviation of Kendall’s *τ* for each EWS that is invariant to changes in the windows size; as well as the associated percentage of trends that are statistically significant.

**Table 1:** Trends for spatially informed EWS invariant to changes in window size.

	SLDS			SPDE			Harvest			Transect-5	
EWS	$\overset{―}{\tau }$	std	$\tau_{sig}$ (%)	$\overset{―}{\tau }$	std	$\tau_{sig}$ (%)	$\overset{―}{\tau }$	std	$\tau_{sig}$ (%)	*τ*	*p*-value
Spatial variance	0.332	0.052	100	0.505	0.027	100	0.635	0.064	100	0.156	0.207
Spatial skewness	0.003	0.043	15	-0.011	0.032	11	-0.001	0.109	9	-0.137	0.178
Spatial correlation	0.739	0.026	100	0.632	0.023	100	0.861	0.030	100	0.565	0.001

Mean and standard deviation of Kendall’s *τ* for spatial EWS invariant to changes in the window size for each model. As well as the percentage of simulated data sets which generate EWS with statistically significant trends, denoted $\tau_{sig}$ (%). Transect-5 data set is empirical (has one realisation) hence we present Kendall’s *τ* and its *p*-value instead.

### Airway data sets

Fig 2 shows the results for data sets for two different models (SLDS and SPDE) of the same physiological system. It can be clearly seen that the multivariate EWS significantly outperform the temporal EWS for the SPDE model (blue). This is because they have strong trends (*τ* close to 1), that agree with the expected behaviour of the signals (increase indicating the phenomena CSD may be present), furthermore the trends are robust (as they do not vary much as the window size is changed), and they are statistically significant for all window percentages considered. Furthermore, we find for the SPDE model that all temporal EWS (except skewness) have decreasing trends, disagreeing with the expected behaviour indicating that these EWS perform poorly on these data sets. This partially supports that for the SPDE model data sets, the multivariate EWS outperform the temporal EWS. Additionally, when considering the mean *τ* values for the SPDE model contained in Table 1 we see that they are less than those of the multivariate EWS. Based on these results we conclude that the best EWS for the SPDE model data sets are the eigenvalues of the covariance matrix.

From Fig 2 we see that neither category of EWS outperforms another for the SLDS model (red), with the exception of skewness. This is because there is similar robustness and statistical significance for all EWS across the range of considered window percentages. Furthermore, we see that the temporal EWS (except skewness) all display trends disagreeing with their expected behaviour, whereas the multivariate EWS trends agree for approximately half of the window range considered. We find from Table 1 that the spatial correlation performs well (agrees with expected behaviour, strong trend, statistically significant) and conclude that the spatial correlation and skewness perform well for these data sets.

In Fig 2 we have displayed the results for the two models to show that EWS behave differently for different mathematical models of the same system. Recognising this difference is important as commonly used models in the study of spatial EWS are discretised reaction-diffusion systems. Hence we find that we should not extrapolate results for EWS performance based on synthetic data from discretised reaction-diffusion systems; as the same EWS can perform differently on other models of the same system.

### Ecological data sets

Fig 3 displays the results for the harvesting model. From this figure we can immediately see that there is essentially zero spread of the Kendall’s *τ* values about their means, for all EWS and across all considered window percentages. This is expected as the lattices used are quite large (100×100). Furthermore, each EWS is robust and statistically significant except the coefficient of the autocorrelation function. From Table 1 we can see that both the spatial variance and spatial correlation perform well too, however the spatial skewness performs poorly for these data sets. Based on trend strength, agreement with expected behaviour, statistical significance and robustness; we cannot conclude that either category of EWS outperforms another as most EWS applied to these data sets perform well.

Fig 4 displays the results for the empirical ecological data set. Immediately we can see that all trends are statistically significant with the exception of the percentage that the leading eigenvalue accounts for the total variation. From Table 1 we see that the trends for both spatial variance and skewness are weak and statistically insignificant. Comparing the results across all EWS for this data set, we see that the largest eigenvalue of the covariance matrix, the standard deviation, and the spatial correlation appear to give the best indication of the transition.

## Discussion

In this study, we have evaluated and compared the performance of spatial and temporal EWS as potential indicators of critical transitions in spatio-temporal systems. We have explored how to evaluate the performance of an EWS to facilitate comparison. From our results, we find that neither category of EWS typically outperforms the other. We can therefore conclude that the practical application of which EWS to use for a given spatio-temporal data set is case dependent. We acknowledge that a complete understanding of why different EWS outperform one another for different systems is beyond the scope of this current work; however, we offer potential explanations for the results obtained, as well as some avenues for future research.

Furthermore, we have demonstrated the following result. It is important to be careful not to overuse and be reliant on SLDS models obtained from discretising reaction-diffusion models when studying EWS. This is because the results obtained for the SPDE model do not necessarily extend to the SLDS model; therefore, we cannot assume that an analysis of EWS for a SPDE model will extend to other spatial models for the same system. Hence, we conclude that over-reliance on reaction-diffusion models as test cases may be misleading and does not extend to other spatial systems.

### Evaluating performance

Previously, measuring the strength of the trend using Kendall’s *τ* has often been used as a quantitative proxy for the performance of EWS. We assess EWS performance using additional factors that must be considered when applying EWS to real world data sets. We have considered how different characteristics of data sets affect which EWS might best provide an indication of critical transitions for a given data set. Characteristics of data sets, such as dimension, resolution, and size, affect which EWS should be used for a given data set. For example, some spatial EWS can be computed from a single snapshot, whereas others require a backwards window in order to be computed. EWS that do not require selecting a window size when they are computed have the benefit that they produce signals of equal length to the data from which they are estimated. This is particularly useful for low-temporal-resolution data sets, such as ecological data sets with few snapshots observed at a low frequency. Whereas for spatial data sets with low-spatial/high-temporal resolution (such as those available in physiology), it is more feasible to use EWS computed using window-based approaches, as there is sufficient data to apply these methods. Hence, the characteristics of the data sets themselves must be considered when applying different EWS. Therefore, in addition to quantitative metrics such as trend strength, statistical significance, and robustness, we recommend considering the qualitative characteristics of data sets and their impact on methodological choices.

### Misleading trends

There are many possibilities why the EWS do not provide indications of critical transitions, even for systems where we know the dynamics and expected outcomes of the EWS. In Fig 2, it can be seen that the variance and autocorrelation indicators for both the SLDS and SPDE models display negative trends for most of the window sizes considered, disagreeing with the expected behaviour of these signals. For each model considered, we know the eigenstructure explicitly. This allows us to understand which modes destabilise when the system undergoes a critical transition, as these are associated with the leading eigenvalues. Furthermore, we know that destabilisation is associated with eigenvalues crossing the imaginary axis, allowing us to infer that CSD is present in the leading modes and therefore measures of CSD should perform well as EWS for transitions in these systems.

We applied generic EWS such as variance and autocorrelation measures to the spatial mean of the SLDS system. We identified unexpected behaviour such as decreasing trends in variance and autocorrelation in the spatial mean for the entire range of the underlying bifurcation parameter prior to the transition.

We have demonstrated that the eigenvalues for the system do not monotonically approach zero from below but rather decrease for 0≤κ<0.6 and then monotonically increase for 0.6<κ. From this behaviour, we would only expect CSD measures to increase for 0.6<κ monotonically. Furthermore, from the linearisation we can only conclude that CSD should be detectable in the eigenmodes of the system, and cannot conclude that it is detectable in the spatial mean. However, we may often only be able to obtain a mean value time series of a system or some other univariate observable; therefore, it is important to understand how low-dimensional EWS behave on univariate data sets of intrinsically high-dimensional systems.

Despite CSD potentially being detectable in the system, we still do not observe increasing trends in the variance and autocorrelation for 0.6<κ. One potential explanation may be that the spatial mean is not an ideal univariate observation for these systems, and effects such as those demonstrated by Morr et al. (where internal noise interference can impact EWS behaviour prior to critical transitions [28]) could be a potential explanation for the misleading trends.

### Future work

One could explore how different univariate observables of intrinsically spatially distributed systems affect the performance of EWS results. Instead of applying temporal EWS to the spatial mean or other average measures, one could apply temporal EWS to the time series obtained from a single location within the spatio-temporal system. However, identifying the ideal location for observing the system with the goal of predicting potential tipping points could be challenging.

Other areas for improving the comparison of indicators lie in changing how the performance of an indicator is assessed. One potential avenue for assessing performance would be to include forewarning times. Determining the forewarning time of signals is complicated, as identifying when a signal becomes statistically significant is challenging, yet ideally should be considered when quantifying the performance of an EWS. In this current work, we have not included this factor in our assessment; however, calculating when a signal becomes statistically significant is possible, and we direct the reader to [29] for more details.

Furthermore, the EWS we have considered are not exhaustive, and could include other options such as the discrete Fourier transform [30], dynamic mode decomposition [6], finite time Lyapunov exponent [31] or patch based indicators such as the patch size distribution [13,32].

## Supporting information

S1 Appendix Derivation of the SPDE model. (PDF)

S2 Appendix Modified Mann-Kendall test. (PDF)

S3 Appendix Model details. (PDF)
