# Supplementary material for: Assessing and comparing early warning signal performance in spatially-structured systems
Source: PLoS One. 2025 Oct 6;20(10):e0332695. doi: 10.1371/journal.pone.0332695 (PMC12500143; doi:10.1371/journal.pone.0332695)
Supplement: S1 Appendix — (PDF) [file pone.0332695.s001.pdf]

Assessing and comparing early warning signal performance in spatially-structured systems

George E. Robinson* and Graham M. Donovan

Department of Mathematics, The University of Auckland, Auckland, New Zealand

* grob767@aucklanduni.ac.nz

Appendix S1: Derivation of the SPDE model

The lattice dynamical system formulated in [1] and the simplified stochastic lattice system derived from it in [2] are both spatial models derived from existing models in the physiology literature [3–5]. Consequently, the term of the stochastic differential equation describing the deterministic dynamics of each element of the lattice is non-linear, with non-linear coupling terms (the contribution of the neighbouring lattice sites to the dynamics are fourth power). This differs from many of the spatial models which are used to study spatial EWS, where the deterministic function consists of a non-linear component adapted from existing models with added diffusion between neighbouring sites. The diffusion is added using the discrete Laplacian term where the contribution of neighbouring lattice sites are linear. This is because many of the SLDS used to study spatial EWS are either existing models with added spatial interactions or discretisations of reaction-diffusion equations, both with added stochastic terms. An example of taking existing models with bistable dynamics and adding spatial dispersion and stochasticity are the spatial ecological models studied by Chen et al. [6]. Examples of papers formulating SPDEs from existing models include [7, 8]. These papers study spatial EWS from a top-down approach by assessing and analysing proposed EWS using simulated data. Another approach to studying spatial EWS are developing potentially useful EWS directly from spatial systems with bistability. Examples of papers which analyse EWS using stochastic partial differential equations include [9–11]. Due to the widespread use of spatial systems modeled using stochastic partial differential equations or their discretisations, we will now derive from the simplified airway SLDS a family of related spatial models resulting in a stochastic reaction-diffusion with similar behaviour to the original model.

We will now make several assumptions and simplifications to derive a family of stochastic partial differential equations from the SLDS studied in [2]. The original motivation for deriving a SPDE from the original SLDS was to apply the EWS studied by Bernuzzi and Kuehn in [9] to spatial systems from lung physiology. To apply the EWS from [9] we must derive an SPDE which has similar behaviour to the original SLDS and is of the form

$$du(x,t) = (\Delta u(x,t) + f(x)u(x,t) - u(x,t)^3)dt + \sigma dW_t. \quad (1)$$

The dimensionless SLDS described in [2] is a simplified model of the SLDS first introduced in [1]. In [1], Donovan and Kritter suggest that simpler models for the radius-pressure can be considered which preserve the qualitative features of the full model. These possibilities include a linear pressure-radius relationship, with parameters extracted from tangency with a non-linear model. They found that an alternative

nonlinear model from Thorpe and Bates [12] and the linear model admit clustered ventilation defects, and therefore Donovan and Kritter conclude that the nonlinearity of the Lambert model is not solely responsible for the behaviour of the model. Therefore we will now further approximate the radius-pressure relationship using a piecewise linear approximation.

Let $R_S(P)$ be used to denote the normalised dimensionless sigmoidal radius-pressure relationship

$$R_S(P) = (1 + \exp(-(P - P_I)))^{-1}. \tag{2}$$

We define the piecewise linear approximation, denoted $R_L(P)$, for the radius-pressure relationship as the first order Taylor approximation to $R_S(P)$ at the inflection point P_I. The function has maximum at $R = 1$ and minimum at $R = r_{min} > 0$. The domain of the linear approximation are the values of P where the linear approximation equals $R = 1$ and $R = r_{min}$. Hence,

$$R_L(P) = \begin{cases} 1, & P_2 < P \\ R'_S(P_I)(P - P_I) + R_S(P_I), & P_1(r_{min}) \leq P \leq P_2 \\ r_{min}, & P < P_1(r_{min}). \end{cases} \tag{3}$$

We define r_{min} as the radius of the airway wall r_w when the luminal radius r_i is closed for a Horsfield order one airway. We computed the value of r_{min} using the following relationship between the airway wall radius and the luminal radius from the supplementary material to the paper by Politi et al. [5]

$$r_w = R_i \sqrt{(1 + \varepsilon_w)^2 + \left(\frac{r_i}{R_i}\right)^2 - 1} \tag{4}$$

where ε_w is the thickness of the airway wall and where R_i is the luminal radius for when the transmural pressure is zero, for a Horsfield order one airway.

For the derivation of the SPDE it is important to distinguish between the homogeneous equilibria of the two lattice dynamical systems obtained by altering the radius-pressure relationship. The first, denoted $r^*_S(\kappa)$, is the root of the lattice dynamical system $\dot{r}_{i,j} = R_S(P) - r_{i,j}$ and is the solution to the following equation

$$\left[1 + \exp\left(\frac{-P_b(0)}{r^4} + \kappa\frac{k}{r} - 5P_b(0)A(1 - r + 1.5(1 - r)^2) + P_I\right)\right]^{-1} - r = 0$$

whereas the other homogeneous equilibrium, denoted $r^*_L(\kappa)$, for the lattice dynamical system $\dot{r}_{i,j} = R_L(P) - r_{i,j}$ is the solution to the equation

$$R'_S(P_I)\left(\frac{P_b(0)}{r^4} - \kappa\frac{k}{r} + 5P_b(0)A(1 - r + 1.5(1 - r)^2) - P_I\right) + R_S(P_I) - r = 0$$

assuming that $P_1 \leq P(r^*_L(\kappa)) \leq P_2$. To derive the SPDE we will begin with the following LDS

$$\dot{r}_{i,j} = R_L(P) - r_{i,j}.$$

The dimensionless function

$$P(r_{i,j}; \mathcal{N}_{i,j}) = P_b - \kappa\frac{k}{r_{i,j}} + P_b A\left(r^4_{i,j} + \sum_{\mathcal{N}_{i,j}} r^4\right)(1 - r_{i,j} + 1.5(1 - r_{i,j})^2) \tag{5}$$

describing the the transmural pressure contains the following local coupling term which describes the restorative forces generated by parenchymal tissue surrounding the airway

$$r^4_{i,j} + \sum_{\mathcal{N}_{i,j}} r^4 = r^4_{i,j} + r^4_{i+1,j} + r^4_{i-1,j} + r^4_{i,j+1} + r^4_{i,j-1}. \tag{6}$$

This highly non-linear local coupling term resembles the discrete two-dimensional Laplacian on an integer lattice $\Lambda \subset \mathbb{Z}^2$ (which is linear),

$$\Delta r_{i,j} = r_{i+1,j} + r_{i-1,j} + r_{i,j+1} + r_{i,j-1} - 4r_{i,j}. \tag{7}$$

We begin by taking a first order Taylor expansion of the coupling terms about the homogeneous equilibrium $r_L^*(\kappa)$,

$$r_{i,j}^4 \approx (r_L^*(\kappa))^4 + 4(r_L^*(\kappa))^3(r_{i,j} - r_L^*(\kappa)). \tag{8}$$

Therefore

$$r_{i+1,j}^4 + r_{i-1,j}^4 + r_{i,j+1}^4 + r_{i,j-1}^4 \approx 4(r_L^*(\kappa))^3 \sum_{\mathcal{N}_{i,j}} r - 12(r_L^*(\kappa))^4 \tag{9}$$

where $\mathcal{N}_{i,j}$ is the set of nearest neighbours of $r_{i,j}$. Adding the term $4(r_L^*(\kappa))^3(-4r_{i,j} + 4r_{i,j})$ (which is equal to zero) to the right hand side gives

$$4(r_L^*(\kappa))^3 \sum_{\mathcal{N}_{i,j}} r - 12(r_L^*(\kappa))^4 = 4(r_L^*(\kappa))^3 \left(\sum_{\mathcal{N}_{i,j}} r - 4r_{i,j} \right) - 12(r_L^*(\kappa))^4 + 16(r_L^*(\kappa))^3 r_{i,j}. \tag{10}$$

Therefore the local coupling term can be approximated as,

$$\sum_{\mathcal{N}_{i,j}} r^4 \approx 4(r_L^*(\kappa))^3 \left(\left(\sum_{\mathcal{N}_{i,j}} r - 4r_{i,j} \right) - 3r_L^*(\kappa) + 4r_{i,j} \right) \tag{11}$$

which contains the two-dimensional discrete-Laplacian $\sum_{\mathcal{N}_{i,j}} r - 4r_{i,j}$. We will now substitute this approximation of the local coupling term into the LDS, $\dot{r}_{i,j} = R_L(P) - r_{i,j}$, and take the continuum limit. Specifically, for the following substitution we will only consider the linear component of the piecewise approximation of the radius-pressure relationship for values of P in the interval $P_1 \leq P \leq P_2$. This is because $R_L(P)$ is dependent on P only on the interval $P_1 \leq P \leq P_2$. For values of P in the domain which are outside of this interval, the function $R_L(P)$ is independent of P and therefore will be independent of the Laplacian term. Expanding $\dot{r}_{i,j} = R_L(P) - r_{i,j}$ gives,

$$\dot{r}_{i,j} = R_S'(P_I)(P - P_I) + R_S(P_I) - r_{i,j}.$$

Substituting the expression for pressure from Equation (5) gives,

$$\dot{r}_{i,j} = R_S'(P_I) \left(P_b - \kappa \frac{k}{r_{i,j}} + P_b A \left(r_{i,j}^4 + \sum_{\mathcal{N}_{i,j}} r^4 \right) (1 - r_{i,j} + 1.5(1 - r_{i,j})^2) - P_I \right) + R_S(P_I) - r_{i,j}.$$

Then substituting the approximation for $\sum_{\mathcal{N}_{i,j}} r^4$ from Equation (11) gives,

$$\dot{r}_{i,j} \approx R_S'(P_I)(P_b - \kappa \frac{k}{r_{i,j}} + P_b A \left(r_{i,j}^4 + 4(r_L^*(\kappa))^3 \left(\left(\sum_{\mathcal{N}_{i,j}} r^4 - 4r_{i,j} \right) - 3r_L^*(\kappa) + 4r_{i,j} \right) \right) (1 - r_{i,j} + 1.5(1 - r_{i,j})^2)) + R_S(P_I) - r_{i,j}.$$

Then taking the continuum limit gives,

$$\partial_t r = R'_S(P_I)(P_b - \kappa\frac{k}{r} + P_b A \left(r^4 + 4(r^*_L(\kappa))^3 \left(\Delta r - 3r^*_L(\kappa) + 4r\right)\right)$$
$$(1 - r_{i,j} + 1.5(1 - r_{i,j})^2)) + R_S(P_I) - r. \tag{12}$$

Therefore the lattice dynamical system $\dot{r}_{i,j} = R_L(P) - r_{i,j}$ defined on a square lattice $\Lambda \subset \mathbb{Z}^2$ can be approximated using the following system of PDEs describing the evolution of a function $r(x,t)$ where $x \in \mathbb{R}^2$,

$$\partial_t r(x,t) = \begin{cases} 1 - r(x,t), & P_2 < P \\ D(r(x,t))\Delta r(x,t) + f(r(x,t)), & P_1 \le P \le P_2 \\ r_{min} - r(x,t), & P < P_1 \end{cases} \tag{13}$$

where the diffusion coefficient is

$$D(r(x,t)) = R'_S(P_I)P_b A \left(1 - r + 1.5(1-r)^2\right) 4(r^*_L(\kappa))^3 \tag{14}$$

and the reaction term is described as

$$f(r(x,t)) = R'_S(P_I)\left[P_b - \kappa\frac{k}{r} + P_b A\left(1 - r + 1.5(1-r)^2\right)\left(r^4 + 4(r^*_L(\kappa))^3(4r - 3r^*_L(\kappa))\right)\right.$$
$$\left. - P_I\right] + R_S(P_I) - r. \tag{15}$$

The system of PDEs described in Equation (13) can be modified by introducing diffusion at each point in the spatial domain regardless of the value of P. Under this assumption that the diffusion is independent of P we obtain the following reaction-diffusion equation with a piecewise reaction function,

$$\partial_t r(x,t) = D(r(x,t))\Delta r(x,t) + \begin{cases} 1 - r(x,t), & P_2 < P \\ f(r(x,t)), & P_1 \le P \le P_2 \\ r_{min} - r(x,t), & P < P_1. \end{cases} \tag{16}$$

A simplified reaction-diffusion equation with a piecewise reaction function which has similar behaviour can be obtained by assuming the the diffusion coefficient is equal to one for all x and t, and is

$$\partial_t r(x,t) = \Delta r(x,t) + \begin{cases} 1 - r(x,t), & P_2 < P \\ f(r(x,t)), & P_1 \le P \le P_2 \\ r_{min} - r(x,t), & P < P_1. \end{cases} \tag{17}$$

Simplifying further we obtain the following reaction-diffusion equation which is independent of the value of P,

$$\partial_t r(x,t) = \Delta r(x,t) + f(r(x,t)). \tag{18}$$

This reaction-diffusion equation maintains similar behaviour to the original SLDS before the bifurcation, however removing the piecewise components in the reaction function removes the alternative stable states after the bifurcation. It is important to mention

that the derivations above assume that P_b is non-constant to maintain total flow. In the discrete case the term for the base pressure is

$$P_b(t) = \frac{P_b(0)N^2}{\sum_{i,j \in \mathcal{L}} r_{i,J}^4}.$$

In the continuum limit this is replaced with

$$P_b(t) = \frac{P_b(0)N^2}{\int_\Omega r(x,t)^4 dx} \tag{19}$$

where $\Omega \subset \mathbb{R}^2$ is a square region of length N.

References

1. Donovan GM, Kritter T. Spatial pattern formation in the lung. Journal of mathematical biology. 2015;70(5):1119–1149.

2. Donovan GM, Brand C. Spatial early warning signals for tipping points using dynamic mode decomposition. Physica A: Statistical Mechanics and its Applications. 2022;596:127152.

3. Horsfield K, Dart G, Olson DE, Filley GF, Cumming G. Models of the human bronchial tree. Journal of applied physiology. 1971;31(2):207–217.

4. Lambert RK, Wilson TA, Hyatt RE, Rodarte JR. A computational model for expiratory flow. Journal of applied physiology. 1982;52(1):44–56.

5. Politi AZ, Donovan GM, Tawhai MH, Sanderson MJ, Lauzon AM, Bates JH, et al. A multiscale, spatially distributed model of asthmatic airway hyper-responsiveness. Journal of theoretical biology. 2010;266(4):614–624.

6. Chen S, O'Dea EB, Drake JM, Epureanu BI. Eigenvalues of the covariance matrix as early warning signals for critical transitions in ecological systems. Scientific reports. 2019;9(1):2572.

7. Guttal V, Jayaprakash C. Spatial variance and spatial skewness: leading indicators of regime shifts in spatial ecological systems. Theoretical Ecology. 2009;2:3–12.

8. Dakos V, Kéfi S, Rietkerk M, Van Nes EH, Scheffer M. Slowing down in spatially patterned ecosystems at the brink of collapse. The American Naturalist. 2011;177(6):E153–E166.

9. Bernuzzi P, Kuehn C. Bifurcations and Early-Warning Signs for SPDEs with Spatial Heterogeneity. Journal of Dynamics and Differential Equations. 2023; p. 1–45.

10. Gowda K, Kuehn C. Early-warning signs for pattern-formation in stochastic partial differential equations. Communications in Nonlinear Science and Numerical Simulation. 2015;22(1-3):55–69.

11. Kuehn C, Romano F. Scaling laws and warning signs for bifurcations of SPDEs. European Journal of Applied Mathematics. 2019;30(5):853–868.

12. Thorpe CW, Bates JH. Effect of stochastic heterogeneity on lung impedance during acute bronchoconstriction: a model analysis. Journal of Applied Physiology. 1997;82(5):1616–1625.
