# Supplementary material for: Assessing and comparing early warning signal performance in spatially-structured systems
Source: PLoS One. 2025 Oct 6;20(10):e0332695. doi: 10.1371/journal.pone.0332695 (PMC12500143; doi:10.1371/journal.pone.0332695)
Supplement: S2 Appendix — (PDF) [file pone.0332695.s002.pdf]

Assessing and comparing early warning signal performance in spatially-structured systems

George E. Robinson* and Graham M. Donovan

Department of Mathematics, The University of Auckland, Auckland, New Zealand

* grob767@aucklanduni.ac.nz

Appendix S2: Modified Mann-Kendall test details

Kendall's τ is related to the S-value according to

$$\tau = \frac{2S}{n(n-1)} \tag{1}$$

where n is the number of observations [1]. The S-value is the number of concordant pairs minus the number of discordant pairs of observations and τ is the normalised S-value achieved by dividing S by the total number of pairs possible from a set of size n. In most cases the z-statistic used to compute the p-value is given by $S/\sqrt{\text{var}(S)}$, however the distribution for S is discrete as given a specific set of observations the values of S can only take on integer values [1]. Furthermore the distribution only tends to Gaussian for large n, hence we use the z-statistic which accounts for the continuity error in the distribution of S given by

$$z = \begin{cases} \frac{S-1}{\sqrt{\text{var}(S)}}, & S > 0 \\ 0, & S = 0 \\ \frac{S+1}{\sqrt{\text{var}(S)}}, & S < 0. \end{cases} \tag{2}$$

Furthermore we use the variant of the modified Mann-Kendall where neither concordant or discordant pairs, otherwise known as ties, are taken into account as these affect the distribution [1, 2]. It is worth pointing out that for computing the significance of EWS, it often the case that ties do not occur and therefore it is not necessary to account for tied observations. Hamed and Rao [3] recommend detrending the time series before computing the autocorrelations of the ranks to avoid insignificant values of $\rho_S(i)$ having an adverse affect on the variance of either S or τ. Yue and Wang [2] suggest that the trend, if it exists, must be removed to avoid adverse affects, and suggest that if the trend is non-linear then an appropriate detrending method to capture the non-linearity should be used. In our case as we expect monotonic trends before transitions within the EWS, that removing linear trends is to be sufficient. This is achieved by using the detrending method used by Yue and Wang which is the Thiel-Sen estimator. More work could be done in this area exploring removing non-linear trends from EWS, if they exist, to determine the statistical significance of potential trends. We have implemented the modified Mann-Kendall test by incorporating the MATLAB code available at this GitHub repository
`https://github.com/atharvaaalok/Modified-MannKendall-Test`.

The modified Mann-Kendall test assumes the null hypothesis that there is no trend in the data and that the distribution of Kendall's τ should be normally distributed with expected value of zero and variance

$$\mathrm{var}(\tau) = \frac{2(2n+5)}{9n(n-1)} \left(1 + \frac{2}{n(n-1)(n-2)} \sum_{i=1}^{n-1} (n-i)(n-i-1)(n-i-2)\rho_S(i) \right) \quad (3)$$

where $\rho_S(i)$ is the autocorrelation at lag i of the ranks of the time series and n is the length of the time series. We can then use the variance of τ to reject the null hypothesis if the p-value is less than a predetermined significance level α. We have chosen α to be 0.05.

References

1. Kendall MG. Rank correlation methods. Griffin; 1948.
2. Yue S, Wang C. The Mann-Kendall test modified by effective sample size to detect trend in serially correlated hydrological series. Water resources management. 2004;18(3):201–218.
3. Hamed KH, Rao AR. A modified Mann-Kendall trend test for autocorrelated data. Journal of hydrology. 1998;204(1-4):182–196.
