# Supplementary material for: Assessing and comparing early warning signal performance in spatially-structured systems
Source: PLoS One. 2025 Oct 6;20(10):e0332695. doi: 10.1371/journal.pone.0332695 (PMC12500143; doi:10.1371/journal.pone.0332695)
Supplement: S3 Appendix — (PDF) [file pone.0332695.s003.pdf]

Assessing and comparing early warning signal performance in spatially-structured systems

George E. Robinson* and Graham M. Donovan

Department of Mathematics, The University of Auckland, Auckland, New Zealand

* grob767@aucklanduni.ac.nz

Appendix S3: Model Details

Here we present the details of the three models used to generate the data sets analysed in our study. We also present the parameter values and their descriptions below. In each model we use a stochastic fast-slow framework as presented by Kuehn [1]. This is achieved by incorporating the underlying bifurcation as a state variable of the slow subsystem and letting it evolve linearly with time. The fast-subsystem dynamics in each case are described by the previously studied models which we have incorporated.

Airway SLDS model

The first model used is a fast-slow stochastic lattice dynamical system (SLDS) adapted from the model derived by Donovan and Brand [2]; which was in turn derived from a previous model by Donovan and Kritter [3]. This system describes a lattice of coupled terminal airway units $r_{i,j}$. Let $r_{i,j}$ denote an arbitrary state variable of a system of coupled elements on a lattice $\mathcal{L}$ where $i, j \in \{1, \ldots, N\}$ and where N is some integer. The fast dynamics are described as follows

$$dr_{i,j} = \left(\left[1 + \exp(-(P_{i,j} - P_I))\right]^{-1}\right)dt + \sigma dW_{i,j} \tag{1}$$

where

$$P_{i,j} = P_b - \kappa\frac{k}{r_{i,j}} + P_b A(1 - r_{i,j} + 1.5(1 - r_{i,j})^2)(r_{i,j}^4 + r_{i-1,j}^4 + r_{i+1,j}^4 + r_{i,j-1}^4 + r_{i,j+1}^4) \tag{2}$$

and where

$$P_b = \frac{P_b(0)N^2}{\sum_{i,j\in\mathcal{L}} r_{i,j}^4} \tag{3}$$

and $\mathcal{L}$ denotes all lattice sites. The slow dynamics are given by $d\kappa = \varepsilon dt$, where $0 < \varepsilon \ll 1$. The parameters values used and their descriptions can be found in Table 1.

Airway SPDE model

The second model used has been derived from the airway SLDS model [2] to obtain a reaction-diffusion model of similar form to those previously used for generating data sets to study EWS. The deterministic fast dynamics are described using the following reaction-diffusion equation

$$\partial_t r(x,t) = \Delta r(x,t) + f(r(x,t), \kappa) \tag{4}$$

Table 1. Airway SLDS parameters and values

Parameter	Definition	Value
$r_{i,j}$	**State variable:** Terminal airway radii at site (i, j)	
κ	**Bifurcation parameter:** Smooth airway muscle pressure	$\kappa \in [0, 1.2]$
ε	Timescale separation	0.01
σ	Standard deviation of additive white noise process	0.01
P	Total pressure on airway wall at site (i, j)	
P_I	Inflection point of sigmoidal airway-pressure relationship (Equation 1)	0.9643
A	Coupling strength	0.6077
k	Bifurcation normalisation parameter	14.065
$P_b(0)$	Global coupling term at $t = 0$	7.25
N	Length of lattice	20

where (dropping the explicit dependence in notation on x and t for clarity),

$$f(r,\kappa) = R'_S(P_I)\Bigg[P_b - \kappa\frac{k}{r} + P_b A\Bigg(1 - r + 1.5(1-r)^2\Bigg) \Bigg(r^4 + 4(r^*_L(\kappa))^3(4r - 3r^*_L(\kappa))\Bigg) - P_I\Bigg] + R_S(P_I) - r, \tag{5}$$

and where

$$P_b(t) = \frac{P_b(0)N^2}{\int_\Omega r^4(x,t)dx} \tag{6}$$

and where $\Omega \subset \mathbb{R}^2$ is a square region with side length N.

We have used $R_S(P)$ to denote the sigmoidal radius-pressure relationship described in Equation 1. We have used $r^*_L(\kappa)$ to denote the homogenous equilibrium of a linear radius-pressure relationship[1]; where the linear relationship is a piecewise linear fitted approximation of the original sigmoidal function. To produce data sets we first discretise the system by defining a lattice of state variables $r_{i,j}$ as before in the intrinsically discrete airway SLDS model, and discretise the Laplacian in two dimensions with diffusion coefficient D giving

$$dr_{i,j} = (f(r_{i,j},\kappa) + D(r_{i+1,j} + r_{i-1,j} + r_{i,j+1} + r_{i,j-1} - 4r_{i,j}))dt + \sigma dW_{i,j}. \tag{7}$$

The slow dynamics are given by $d\kappa = \varepsilon dt$, where $0 < \varepsilon \ll 1$, as before. Unless otherwise specified, the parameter values are the same as in the airway SLDS model contained in Table 1, additional parameters are contained in Table 2.

Table 2. Airway SPDE parameters and values

Parameter	Definition	Value
$R_S(P_I)$	Value of the sigmoidal radius-pressure relationship evaluated at P_I	0.5
$R'_S(P_I)$	Value of the derivative of the function $R_S(P)$ evaluated at P_I	0.25
D	Diffusion coefficient	1

Harvesting model

The third model is a well studied spatially extended ecological model [4–7] which describes a spatially distributed biomass under harvesting. The dynamics of the biomass

[1]The expression for the linear radius-pressure relationship is contained in Appendix S1.

at each lattice site are described using the following stochastic lattice dynamical system

$$dX_{i,j} = (f(X_{i,j}, c) + D(X_{i+1,j} + X_{i-1,j} + X_{i,j+1} + X_{i,j-1} - 4X + i, j))dt + \sigma dW_{i,j} \quad (8)$$

where the deterministic dynamics $f(X_{i,j}, c)$ are described using the following form with a logistic growth term and loss due to grazing pressure

$$f(X_{i,j}, c) = X_{i,j}\left(1 - \frac{X_{i,j}}{K}\right) - c\frac{X_{i,j}^2}{X_{i.j}^2 + 1}. \quad (9)$$

The original dynamics [4] were previously adapted by defining a lattice of spatially distributed state variables $X_{i,j}$ with added diffusion described by the discretised Laplacian in two dimensions with diffusion coefficient D, resulting in the model used by Chen et al. [6] and Dakos et al. [7] from whom we have taken the parameter values contained in Table 3. As with all models used in this study we have used the fast-slow framework presented by Kuehn [1] where the state variables $X_{i,j}$ form the fast subsystem with the underlying bifurcation parameter c evolving at a much slower timescale with timescale separation ε.

Table 3. Harvest Model parameters and values

Parameter	Definition	Value
$X_{i,j}$	**State variable:** Quantity of biomass at lattice site (i, j)	
c	**Bifurcation parameter:** Grazing pressure	$c \in [1, 3]$
K	Carrying capacity	10
D	Diffusion coefficient	0.2
σ	Standard deviation of additive white noise process	0.1
ε	Timescale separation	0.001

References

1. Kuehn C. A mathematical framework for critical transitions: Bifurcations, fast–slow systems and stochastic dynamics. Physica D: Nonlinear Phenomena. 2011;240(12):1020–1035.

2. Donovan G, Brand C. Spatial early warning signals for tipping points using dynamic mode decomposition. Physica A: Statistical Mechanics and its Applications. 2022;596:127152.

3. Donovan GM, Kritter T. Spatial pattern formation in the lung. Journal of mathematical biology. 2015;70(5):1119–1149.

4. May RM. Thresholds and breakpoints in ecosystems with a multiplicity of stable states. Nature. 1977;269(5628):471–477.

5. Dakos V, Carpenter SR, Brock WA, Ellison AM, Guttal V, Ives AR, et al. Methods for detecting early warnings of critical transitions in time series illustrated using simulated ecological data. PloS one. 2012;7(7):e41010.

6. Chen S, O'Dea EB, Drake JM, Epureanu BI. Eigenvalues of the covariance matrix as early warning signals for critical transitions in ecological systems. Scientific reports. 2019;9(1):2572.

7. Dakos V, van Nes EH, Donangelo R, Fort H, Scheffer M. Spatial correlation as leading indicator of catastrophic shifts. Theoretical Ecology. 2010;3:163–174.
